# Multiple phase transitions in an agent-based evolutionary model with neutral fitness

**DOI:** 10.1098/rsos.170005

**Published:** 2017-04-12

**Authors:** Dawn M. King, Adam D. Scott, Sonya Bahar

**Affiliations:** 1 Department of Physics and Astronomy and Center for Neurodynamics, University of Missouri at St Louis, St Louis, MO 63121, USA; 2 Division of Oncology, Department of Medicine, Washington University, St Louis, MO 63108, USA; 3 McDonnell Genome Institute, Department of Medicine, Washington University, St Louis, MO 63108, USA

**Keywords:** phase transition, extinction, agent-based model, neutral theory, clustering, speciation

## Abstract

Null models are crucial for understanding evolutionary processes such as speciation and adaptive radiation. We analyse an agent-based null model, considering a case without selection—neutral evolution—in which organisms are defined only by phenotype. Universal dynamics has previously been demonstrated in a related model on a neutral fitness landscape, showing that this system belongs to the directed percolation (DP) universality class. The traditional null condition of neutral fitness (where fitness is defined as the number of offspring each organism produces) is extended here to include equal probability of death among organisms. We identify two types of phase transition: (i) a non-equilibrium DP transition through generational time (i.e. survival), and (ii) an equilibrium ordinary percolation transition through the phenotype space (based on links between mating organisms). Owing to the dynamical rules of the DP reaction–diffusion process, organisms can only sparsely fill the phenotype space, resulting in significant phenotypic diversity within a cluster of mating organisms. This highlights the necessity of understanding hierarchical evolutionary relationships, rather than merely developing taxonomies based on phenotypic similarity, in order to develop models that can explain phylogenetic patterns found in the fossil record or to make hypotheses for the incomplete fossil record of deep time.

## 1. Introduction

Neutral theories of ecology and of evolution via genetic drift have been contentious topics among evolutionary biologists[1–3], owing to these theories’ assumption that selective forces may not need to act upon fitness differences between organisms [4] or genomes [5,6] in order to drive ecological community structure or the formation of species. Neutral theory, however, has continued to thrive in the academic literature [7,8], and it can be a useful tool for uncovering underlying mechanisms of evolutionary processes. In particular, its emergence in the agent-based modelling community has unravelled the minimal necessary mechanisms for speciation to occur, while providing a null hypothesis against which models of selection can be tested. However, many aspects of neutral, null models remain unexplored, which has hindered the development of null hypotheses that can classify ecological models based on the underlying dynamical rules of the system [9,10]. For example, in a 2005 review, DeAngelis & Mooij [11] categorized more than 900 agent-based models of ecological and evolutionary processes into seven major types (such as collective motion, foraging, population dynamics and speciation). In contrast with the widespread acceptance among population geneticists of genetic drift as a meaningful baseline model, many of the ecological models categorized by DeAngelis & Mooij did not investigate neutral evolution.

Understanding the evolutionary process of speciation involves identifying which mechanisms yield observed patterns of biological disparity (number of distinct morphologies, phenotypes or body plans within a population) and diversity (number of distinct species or groupings of biological taxa in a population). The latter has been the focus of much agent-based work [12–14]. Because many studies have not incorporated evolutionary mechanisms into their models of extinction risk [15], there is no clear understanding of how a dynamically evolving population can re-evolve following a mass extinction. With mounting evidence that Earth is in the midst of the sixth major extinction [16,17], now formally proposed to be renamed the Anthropocene Epoch, the drastically increased extinction risk for many species highlights the urgency of investigating mechanisms of evolution following a mass extinction [18]. A possible first step to accomplish this is to understand the properties of survival-to-extinction phase transitions in a model of evolutionary dynamics.

While sympatric speciation is thought to be a less common mechanism than others (allopatric or parapatric), it is nonetheless a significant driver of evolutionary processes, especially adaptive radiation [19]. Adaptive radiation in sympatry has been observed, for example, in East African cichlid fishes, the Hawaiian silverswords [19], and the *Anolis* lizards of the Caribbean [20]. It is also speculated to occur following an extinction event when biota are cleared from previously occupied niches [21]. While experimental observation shows that sympatric speciation can be significant for natural processes, computational models can address the important mechanisms that drive this form of speciation. For instance, Kondrashov & Kondrashov [22] showed the possibility of sympatric speciation with the addition of sexual selection and a *fitness component* via different implementations of trait choice between the sexes, while Dieckmann & Doebeli [23] used a genetic assortative mating model to demonstrate that competition for similar resources ‘can initiate sympatric speciation even if mating depends on an ecologically neutral marker trait’.

It has been hypothesized that if the underlying mechanisms of pattern and community formation can be understood, then, by observation of how populations fill their niches, the process (or mechanism) by which species evolved can be inferred [24]. Clustering patterns have been demonstrated in evolutionary, agent-based models with distinctly different dynamical rules [9,12,24–30]. For example, Young *et al*. [26] used a diffusion equation to govern population dynamics, while de Aguiar *et al*. [12] implemented an assortative mating scheme that allowed independent organisms to pick mates based on spatial and genetic proximity. Even though the above-cited models used different dynamical processes, they all demonstrated that an essential ingredient for the development of emergent clustering patterns was a spatial asymmetry between the birth and death process. Births must disperse locally, while death is a global affair. While these studies established that there are essential dynamical rules for emergent clustering, there is little consensus (as noted above) over what the appropriate null condition is in agent-based modelling for testing selection theories that relate to evolutionary processes such as speciation or adaptive radiation.

The neutral model designed by de Aguiar *et al.* [12] used a hermaphroditic population that combined both genetic and spatial dynamics to compare simulated spatial patterns (clustering of individuals) to patterns observed in nature. The model predicts a constant speciation rate over long periods of time; after an initial transient period of rapid growth dominated by mutation and recombination, the number of species reached a steady state. Their observations were compared with the mammalian fossil record of the Meade Basin in Kansas, with the striking conclusion that speciation events in the basin occurred without geographical barriers. As their model is inherently sympatric, these results were cited as ‘disproving this once-dominant view, which was held because of the expectation that speciation is promoted by physical barriers’, and as evidence that the observed speciation events were not caused by glaciation, because the simulated clustering patterns, modelled without geographical barriers, mimicked patterns found in the mammalian fossil record. This model is classified as an equilibrium system, because populations were held constant throughout the entire simulation, and thus no form of extinction owing to outside sources was possible [12]. However, because life is inherently a non-equilibrium process (no population may ever come back from extinction), these models are of limited realism. Furthermore, as pointed out by Jablonski [31], until agent-based models are able to show hierarchical relations of evolutionary processes, thus relating a microevolutionary simulation to macroevolutionary processes revealed by the fossil record, comparison with actual biological processes will remain speculative at best.

The possibility of a non-equilibrium transition (e.g. the possibility of an ‘absorbing’ state such as extinction) was incorporated by Chave and co-workers in their spatially explicit model of sessile organisms, with the aim of understanding mechanisms that underlie community formation [24]. They compared the patterns that emerged when offspring were dispersed locally versus globally, and when the underlying dynamics between species was governed by neutral conditions versus models where birth and death rates among the different species were asymmetric with simple trade-offs (for example, species with a lower reproductive rate could compete better for space). They found that the governing mechanism of biodiversity was offspring dispersion, rather than the presence or absence of neutral conditions. This recalls the results cited above, which showed that local dispersion of births and global deaths distributed randomly throughout the population were the key requirements for emergent clustering, yet also highlights the fact that an underlying selective process is not necessary for emergent clustering. This result is also highlighted by a comparison of the work of Dees & Bahar [32], which demonstrated emergent clustering in a rugged fitness landscape, to that of Scott *et al*. [30] who, in a nearly identical model except for the introduction of neutral conditions, also demonstrated emergent clustering. Further, while the studies described above reveal key requirements for cluster formation, there have been few computational examinations of patterns of population disparity, with exception of Raup & Gould [33], Slowinski & Guyer [34], and Pie & Weitz [9]. This leaves an important aspect of biological pattern formation by the wayside—an aspect central to important problems of adaptive radiation, such as the re-investigation of the Burgess Shale fossils, where high population disparity and low species diversity prevail [21,35].

Previous work using an agent-based model of evolutionary dynamics similar to the work presented below, but on a rugged fitness landscape, exhibited a phase transition from survival to extinction as a single control parameter was varied [32]. The control parameter, mutability (*µ*), characterized how phenotypically different offspring could be from their parent organisms, which reproduced via an assortative mating scheme. It was then shown that this phase transition behaviour transpired even with implementation of a neutral fitness landscape, and not only for the assortative mating reproduction scheme, but also for bacteria-like fission [30]. Continuous, absorbing phase transition behaviour was shown for both reproduction schemes and for two order parameters, the population size and the number of clusters (considered analogous to species). Consistent with the conclusion of Chave *et al.* [24] that selective forces do not determine the emergent properties of the system, random mating was also investigated in the neutral version of this model, and in this case an inherently different type of population-based phase transition—a non-continuous one—was observed, and no clustering behaviour occurred. A similar result that showed a lack of ‘evolutionary branching’ with random mating was presented by Dieckmann & Doebeli [23]. Most recently, it was shown that this survival-to-extinction phase transition belongs to the directed percolation (DP) universality class, thus classifying and demonstrating universality in a neutral model of evolutionary dynamics [36].

In this paper, we, specifically, investigate the DP phase transition behaviour as a function of a new control parameter, the individual death probability *δ*, and examine the specific organismal patterns that arise in the survival regime on the morphospace. The model is neutral in that no organism has a selective advantage over another and all organisms are subjected to the same dynamical rules. Using this null model, we investigate the relationship between morphological and lineage diversity. This relationship is of particular importance for palaeontologists who must rely on morphological character alone to trace ancestry in the fossil record of deep time [9]. This long-standing problem of taxonomic classification was highlighted in the seminal paper by Raup & Gould, who used branching random walks to investigate lineages [33]. They showed that within a species of known lineage morphological ‘outliers’ could occur, and thus computationally identified a potential problem with characterizing species based on morphology alone.

The agent-based model used here incorporates the fundamental characteristics of Darwinian evolution (heritability, variation and competition), but on a neutral fitness landscape. The model can be characterized by a branching and coalescing random walk, a mathematical process that directly maps onto a reaction–diffusion (RD) process in physics. The RD process A → 2A, A + A → A, and A → Ø can undergo a continuous phase transition belonging to the DP universality class [37–39]. This corresponds to the birth of new offspring (A → 2A), coalescence resulting from competition between two individuals leading to the death of one of them (A + A → A), and random death (A → Ø).

We specifically investigate emergent properties, namely, the DP phase transition behaviour that occurs when individual agents reproduce, mutate and die on generational timescales that, for most species, could not be typically observed in human lifetimes. We show that two types of phase transition occur in this model: a non-equilibrium, absorbing transition belonging to the (2 + 1) dimensional DP universality class (where the terminology refers to two spatial and one temporal dimension) and an equilibrium transition that could allow for the investigation of morphological disparity patterns. Note that a non-equilibrium, absorbing transition means that the system has a state from which it can never escape (i.e. extinction), while in an equilibrium transition the population can move in either direction between the two states (i.e. from an aggregated state to a uniform population distribution) with an equal probability.

## 2. Methods

Simulations took place on a two-dimensional, continuous phenotype space with finite boundaries, with 45 arbitrary units along each axis. Therefore, the description of individuals is based on a continuous spectrum of phenotypic traits rather than on their location in geographical space. Each simulation started with an initial uniformly randomly distributed population of 300 individuals. While this limits the analogy between our model and sympatric speciation, these initial conditions were selected in order to minimize the transient time for populations to reach a steady state. The number of initial organisms has no effect on the phase transition behaviour of the system [40]. In fact, as the dynamics of our system behaves as a Markov process, with each state depending only on the prior state, the distribution of organisms at any given generation could be taken as an initial condition for future generations.

In order to mimic neutral evolution, the fitness landscape was held constant, meaning that each organism produced the same number of offspring (denoted by the fitness *f*). The behaviour of the system was investigated as a function of fitness and the individual death probability *δ*. When *δ* served as the control parameter, the fitness was set to *f* = 2, and each simulation lasted for 2000 generations unless extinction occurred first. When the system's behaviour was investigated as a function of fitness, the individual death probability was held constant at *δ* = 1%, and each simulation was performed for 250 generations.

### 2.1. Reproduction schemes

Reproduction occurred via one of three schemes. *Random mating* is considered as a control condition; in this case, each organism randomly chose a mate and an ‘alternate’ mate from the general population. In *assortative mating*, each organism chose its nearest neighbour as its mate, and its second nearest neighbour was identified as an ‘alternate’ mate. The alternate mate is relevant to how clusters were determined (discussed below). Note that in both the random and assortative mating schemes, mating pairs need not be monogamous. The third scheme, *asexual reproduction*, is a branching process in which each organism splits into two offspring.

### 2.2. Offspring dispersal

**Figure 1.**
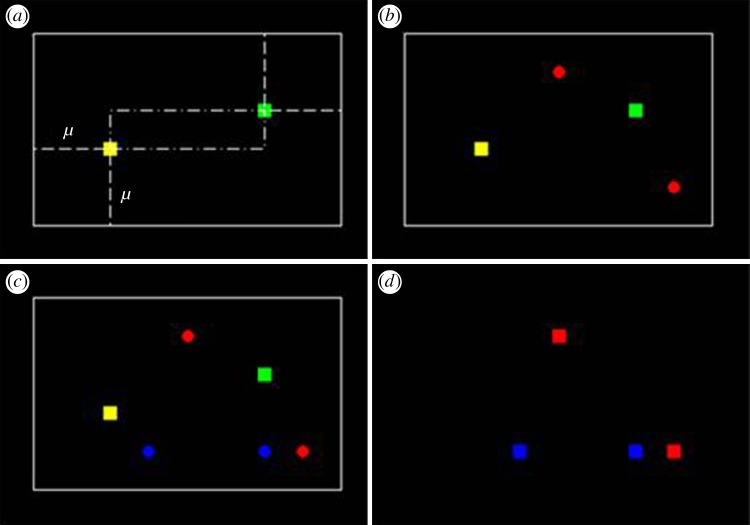
Schematic diagram for assortative mating. Steps (*a–d*) can be read from left to right. Parents are labelled as squares and offspring as circles. (*a*) One reference organism (yellow) selects its nearest neighbour (green) as a mate. Offspring are distributed in an area defined by the locations of the two parent organisms, extended by the mutability *μ*. (*b*) Yellow's offspring organisms are generated (red circles). (*c*) Green's offspring are generated (blue circles). Note that this example assumes that the yellow parent organism is also the nearest neighbour of the green organism; this will not always be the case, and thus mating pairs will not necessarily be ‘monogamous’. (*d*) After every parent has mated (each acting once as the reference organism), all parents are removed, leaving their offspring to act as parents for the next generation.

Offspring organisms were dispersed in each simulation depending on the fitness landscape and the mutability *µ*. For assortative and random mating, offspring were uniformly randomly dispersed in an area around the parents, limited by *µ* (illustrated in figure 1 for assortative mating). For asexual reproduction, new offspring were distributed randomly in a 2µ*2*µ* area centred at the parent organism. The fitness, *f*, determined how many offspring were placed in the dispersal area of the parent organism(s). The system was investigated at *µ* = 0.30, 0.60 and 0.90.

### 2.3. Death processes

Generations do not overlap, and thus parent organisms were removed after offspring production. The offspring then endured a series of death processes, implemented in the following order. First, based on a competition limit *κ*, we randomly removed one of two organisms when neighbouring within a radial distance of *κ* = 0.25 units. This corresponds to competition for resources between phenotypically similar organisms living in the same niche. The value of *κ* was held constant for all simulations shown here, but could in principle be used as another control parameter to drive the system dynamics. Other vagaries of fate such as natural disaster or poor environmental conditions were represented by a stochastic death process where each organism was assigned an individual death probability *δ*, which served as a system control parameter and was varied on the interval [0.01, 0.50] in increments of 0.01. Each individual in every generation was randomly assigned a number between 0 and 1, and any organism with a value equal to or below *δ* was removed. Lastly, any organism found outside the boundary of the landscape was removed. Finite boundary conditions can be interpreted as necessary environmental selective pressures to counteract processes such as Fisherian runaway [41].

### 2.4. Cluster formation

**Figure 2.**
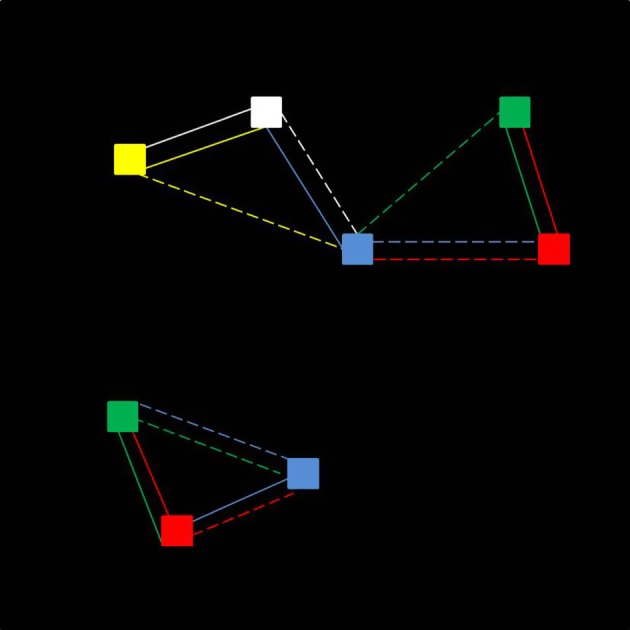
Schematic representation of the formation of reproductively isolated clusters. This algorithm is used for both assortative mating and asexual reproduction. The nearest organism to a reference organism is its ‘mate’ (solid lines). The second nearest organism to the reference organism is its ‘alternate’ mate (dashed lines). Lines are coloured to indicate the mate and alternate mate of the correspondingly coloured reference organism; for example, the white organism's mate is the blue organism, and its alternate is the yellow organism.

**Figure 3.**
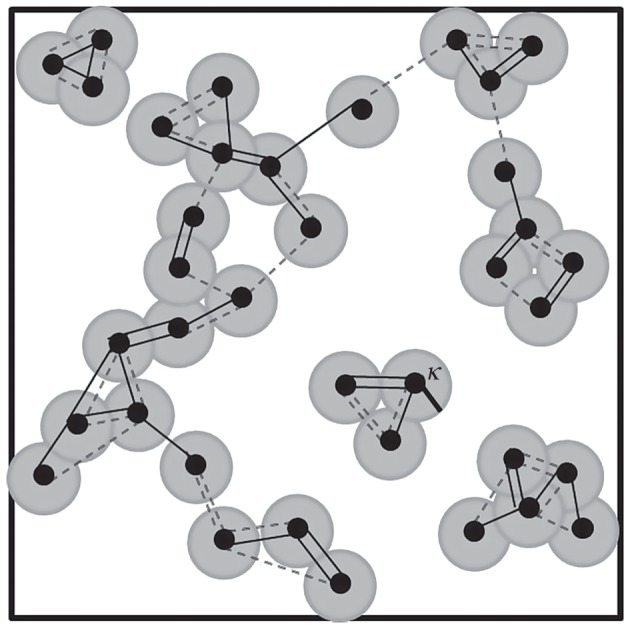
Schematic representation of clusters formed by mate choice and by percolating discs. Dots represent mating organisms. Mates (solid lines) and alternate mates (dashed lines) are included in the definition of a cluster. *κ* = 0.25 for all simulations, and represents the radii of the discs. Four clusters of bonded mates and seven disc clusters are shown. Note how the largest cluster of bonded mates spans the space from end to end, while the discs do not form a continuous overlapping chain. In such a case, bond percolation can occur in the absence of site percolation.

Clusters were determined by mating pairs, which aligns this algorithm with the biological species concept: when a closed set is formed, a reproductively isolated population is obtained. The clustering algorithm is represented schematically in figure 2 for the assortative mating scheme. A group of three organisms—the reference organism, its mate, and its second nearest neighbour, its alternate mate—formed *cluster seeds*, and then an iterative process determined whether organisms within one cluster seed belonged to another. If so, then this other cluster seed was incorporated into the growing cluster. The closed group of mating organisms generated by this process is representative of a species and can be represented as a bonded-cluster of mates (as seen in figure 3). The same algorithm is also used to identify clusters in the asexual reproduction case. However, in this case, the nearest neighbours and second nearest neighbours represent the most phenotypically similar and second-most phenotypically similar organisms, respectively, instead of actual mates. For the random mating scheme, both mates and alternate mates were chosen at random, and the clustering algorithm was applied to these mates as in the assortative case.

## 3. Analysis techniques

### 3.1. Nearest neighbour index, *R*

The Clark and Evans nearest neighbour index, *R*, characterizes ‘the manner and degree to which the distribution of individuals in a population on a given area departs from that of a random distribution’ [42]. The random distribution is defined by the random placement of *N* points on a space. The average nearest neighbour distance in a randomly distributed population is $r_{E}=1\text{/}2\sqrt{\rho }$, where *ρ* is the population density [42]. The ratio *R* = *r_A_*/*r_E_*, where *r_A_* is the actual measurement of the average nearest neighbour distance of the population being sampled, quantifies a population's departure from a random distribution. Thus, when *R* < 1, the population is distributed in a more clumped, aggregated manner, and when *R* > 1, the population is more uniformly dispersed across the space. At *R* = 1, the spatial distribution of the population is said to be random. For the maximum packing of a space (with a population arranged in a hexagonal lattice structure), the measure approaches a limit of *R* = 2.1491 [42].

### 3.2. Continuum percolation

The existing organisms chose mates and cluster on a continuous space. The organisms in these clusters can be represented as discs with radius *κ*, and these discs may overlap up to the radial distance (see figure 3 for schematic representation). In the case of overlapping percolating discs, the percolation threshold is considered to occur at the point for which there is a continuous chain of overlapping discs that spans the space from end to end. In models of this type, the fraction of the landscape filled, which is a function of some probabilistic filling factor [43], can act as the order parameter for the system. For the two-dimensional continuum model with overlapping discs, *ϕ* = 1 − *e*^−*η*^, where *η* = *ρa* is the filling factor, *ρ* is the population density and *a* is the disc area. Critical percolation values have been calculated to be $\eta_{c}≅1.12\;$ and $\phi_{c}≅0.67$ [44].

## 4. Results

**Figure 4.**
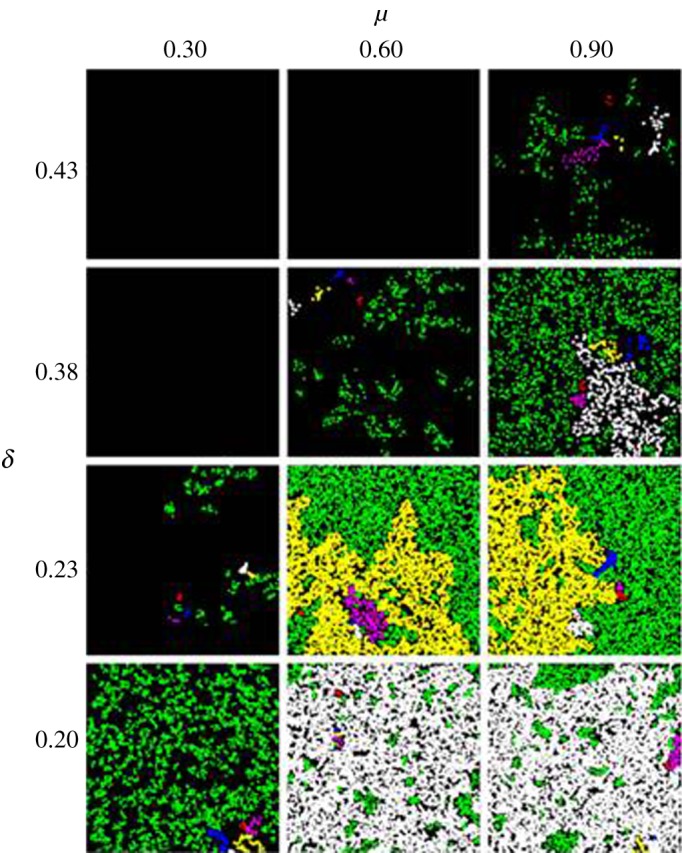
Clustering for assortative mating on a 45 × 45 landscape at 2000 generations. Individuals are represented by dots, with example clusters highlighted in red, white, yellow, purple and blue. The system is shown at critical values of *δ*_c_ = 0.23, 0.38, 0.43, for *μ* = 0.30, 0.60, 0.90, respectively. For *δ* = 0.20, the system exists within the survival regime for each value of *μ* shown.

**Figure 5.**
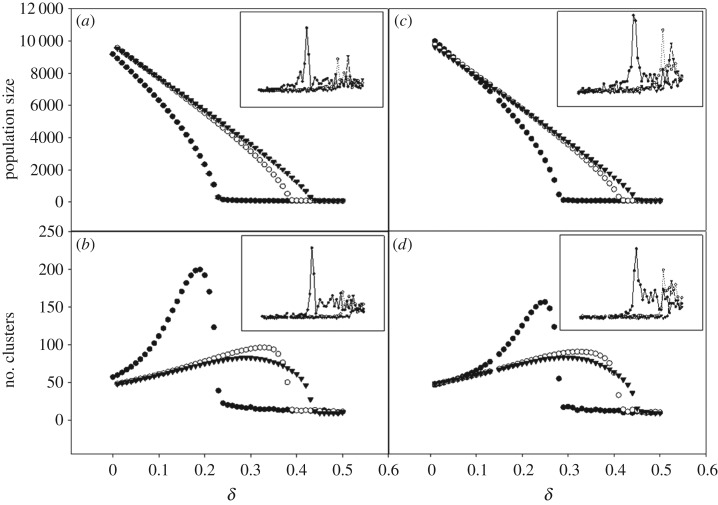
Phase transition behaviour for population size and number of clusters for the assortative mating scheme (*a*,*b*) and asexual reproduction (*c*,*d*). Solid circles represent *μ* = 0.30, hollow circles *μ* = 0.60 and triangles *μ* = 0.90. Insets show a sharp rise in the standard deviation, averaged over five simulations, indicating the critical point of the phase transition. *Y*-axis of insets scaled from (*a*) 0–100 (*b*) 0–60 (*c*) 0–60 (*d*) 0–6; horizontal axes of all insets scale from 0 to 0.6.

Representative clustering for assortative mating is shown in figure 4. The horizontal axes show three different mutabilities (*µ* = 0.30, 0.60 and 0.90), while the vertical axes show various values of the individual death probability, *δ*, sampled above and below the non-equilibrium phase transitions shown in figure 5. The approximate critical values *δ*_c_ (defined here as the first value of *δ* for which the population survives for 2000 generations) for each value of *µ* are also shown in figure 4. Note the qualitative difference in clustering for different values of *δ* and *µ*. Near criticality, clusters are clumped or aggregated, while above criticality, organisms are distributed more uniformly, and the largest cluster of mates can span the landscape from end to end. This indicates that there is a critical point for which a bonded-cluster of mates can span the space. Traditionally, the theory of ordinary percolation defines percolation on a regular lattice, where *site percolation* is defined with respect to the nodes of a given lattice configuration, and *bond percolation* is defined with respect to the bonds connecting the nodes [45]. We adopt similar terminology for the continuous space, considering the organisms as nodes and the interactions between them as bonds. Thus, mate choice creates bonded-clusters of mates. Likewise, when a parent generates offspring this creates a genealogical bond between the parent and offspring nodes.

Absorbing, continuous phase transitions for the order parameters of population size and number of clusters are shown in figure 5, for assortative mating (figure 5*a*,*b*) and for asexual reproduction (figure 5*c*,*d*). The phase transitions are classified as non-equilibrium because the system can enter an absorbing state of extinction; once organisms reach extinction they can never come back. The continuous nature of the phase transitions are demonstrated by the sharp rise in the standard deviation of the order parameter at the critical point, *δ*_c_ (insets in figure 5). For *µ* = 0.30, 0.60 and 0.90, the critical point for assortative mating occurred at approximately *δ*_c_ ≈ 0.23, 0.38 and 0.43 (see also figure 4). For asexual reproduction, critical points occur at *δ*_c_ ≈ 0.26, 0.40 and 0.44.

We find that, as *µ* increases, the system becomes more robust against extinction: for *µ* = 0.90, the population could survive even with a death probability of *δ* = 0.44 (figure 5*a*,*c*). However, with increasing system robustness, there is a significant loss in diversity because the number of clusters drops (as indicated by a decrease in the maximum number of clusters for *µ* = 0.60, 0.90, shown in figure 5*b*,*d*).

Non-critical (or non-continuous) phase transition behaviour was observed as a function of *δ* for the population size (data not shown) in the random mating case. Random mating also resulted in significantly different clustering behaviour than the assortative and asexual reproduction cases because only one cluster was observed in each random mating simulation.

**Figure 6.**
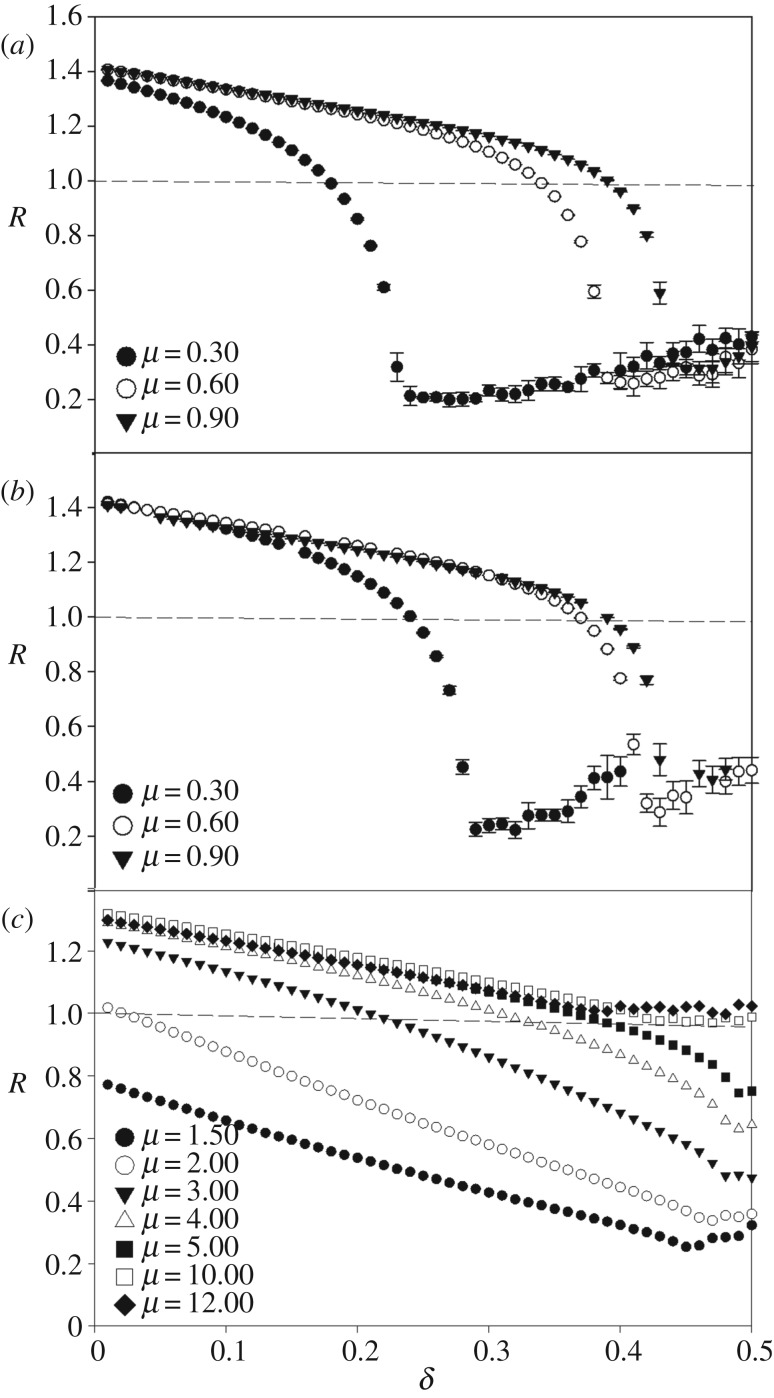
*R* transition curves averaged over five simulations. Assortative mating (*a*) and asexual reproduction (*b*) show continuous phase transitions. The random mating case (*c*) shows no critical phase transition behaviour. Dashed horizontal lines indicate *R* = 1.

**Figure 7.**
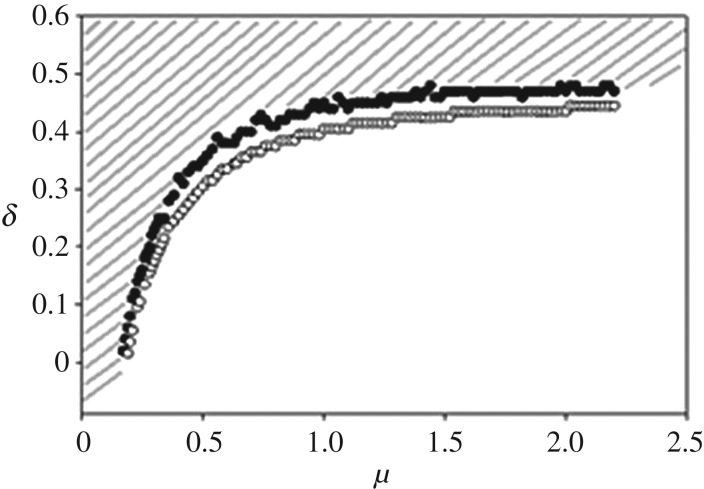
Parameter space plot for assortative mating. Shaded area represents the extinction regime. Filled circles indicate the critical points of the continuous phase transition. Open circles indicate where *R* = 1, thus showing that the populations always change their distribution structure within the active phase of the phase transition.

The clustering behaviour of the organisms can be quantified using the Clark & Evans nearest neighbour index, *R*, as shown in figure 6. This index itself appeared to undergo a continuous phase transition as a function of *δ* for the assortative mating and asexual reproduction cases (figure 6*a*,*b*). As with the population size and cluster number order parameters, a non-critical phase transition occurred in the random mating case (figure 6*c*). The critical points shown for the *R* transition occur at the same values of *δ*_c_ as shown in figure 5. Note, however, that the transition from a clumped, aggregated distribution (*R* < 1), to a random distribution (*R* = 1), to a uniform distribution (*R* > 1) does *not* occur at the critical point of these absorbing phase transitions, and that this distribution change transpires, at least for some parameter values, regardless of the mating scheme. The dashed lines in figure 6 indicate where this distribution change takes place. For the bacterial and assortative schemes, the distribution transition occurs within the active (survival) phase of the extinction/survival phase transitions, while, for the random mating scheme, the transition occurs only for *μ* ≥ 2 and *μ* ≤ 10. For *μ* < 2, *R* < 1; for *μ* > 10, *R* > 1. Figure 7 shows a parameter space plot for the assortative mating scheme, with critical values of *μ*_c_ and *δ*_c_ represented by filled circles, and the values of *µ*_R_ and *δ*_R_ for which *R* = 1 represented by open circles. The *R* transition always exists within the active phase of the phase transition, as the (*µ*_R_, *δ*_R_) curve lies below the (*µ*_c_, *δ*_c_) curve, in the region of parameter space where the system is in its survival phase.

**Figure 8.**
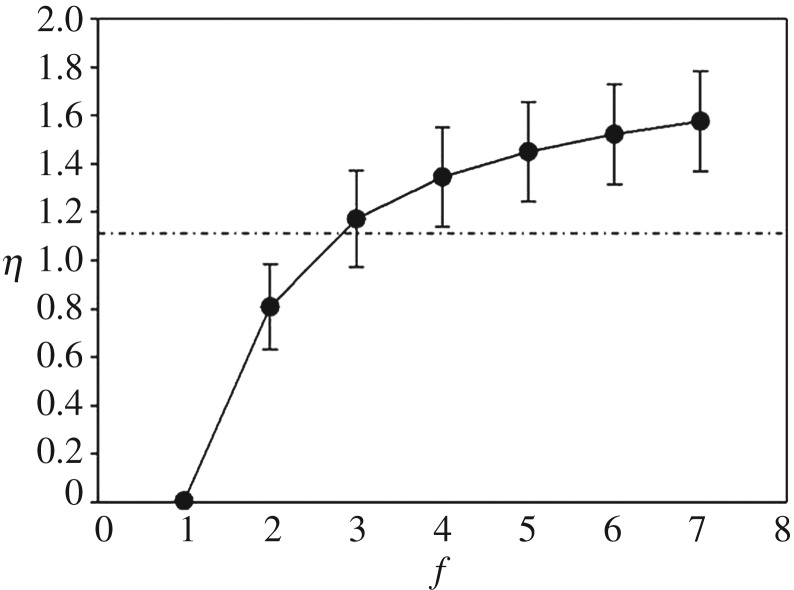
The filling factor for the continuum percolation analysis as a function of fitness. The horizontal dashed line represents the percolation threshold at *η*_c_ ≈ 1.12. Data were averaged over 20 simulations for each fitness value, and simulations were run for 250 generations.

We further investigated the clustering behaviour of the system as a function of the fitness *f* to determine whether a space-filling, continuum cluster of discs can percolate across the space*.* Figure 8 indicates that the critical value of the filling factor *η*_c_ ≈ 1.12 (dashed line) occurs between *f *= 2 and *f *= 3. Thus, at *f *= 2, the value of fitness for which the all of the previous simulations were performed, the continuum percolation threshold has *not* yet been reached. Thus, no continuous cluster of discs existed, yet spanning bonded clusters of mates, defined by nearest-neighbour and next-nearest neighbour bonds, were observed—as indicated by the example spanning clusters shown in figure 4.

## 5. Discussion and conclusion

The existence of non-equilibrium, continuous phase transitions, for both fluctuating and neutral conditions, as a function of mutability has previously been demonstrated in a family of agent-based evolutionary models [30,32,36]. This work has presented a version of the model that incorporates not only neutral fitness conditions but also an individual death probability that is uniform for all organisms at every generation, and we have shown that the existence of a non-equilibrium, continuous phase transition (figure 5) as this parameter is varied.

In general, any model that is in agreement with the DP conjecture, and undergoes an RD process of A → 2A, A + A → A and A → Ø should belong to the DP universality class [37–39]. The phase transition shown here follows the same RD process. Further, it is consistent with the first three properties of the DP conjecture outlined by Henkel *et al.* [38], namely that it: (i) displays a continuous transition from a fluctuating active state (survival) to a unique absorbing state (extinction), (ii) it is characterized by a non-negative one-component order parameter, and (iii) it follows short-ranged dynamical rules.

The fourth part of the DP conjecture requires that a system be free of spatiotemporal disorder. The use of a null model brings us as close to this requirement as possible, given the stochastic nature of the simulation. First, the flatness of the fitness landscape—in other words, the absence of selection—minimizes spatial disorder. As for temporal disorder, previous studies of mutability-driven phase transitions [30,32,36] used a time-dependent death probability, *δ*^τ^, defined such that individuals had up to a 70% chance of being killed in any given generation (i.e. the percentage of deaths varied from generation to generation). Here, instead of *δ*^τ^, we assign an individual death probability, *δ*, so that in each generation every individual has the same probability of being killed. This modification allows for a more stringent matching to the definition of neutral fitness outlined by Hubbell [4], and hence provides a more suitable null condition because all individuals not only had the same number of offspring, but also shared the same probability of death. Subjecting a population to an individual death probability rather than a time-dependent death probability also reduces the temporal disorder in the system, bringing the model into closer agreement with the last requirement of the DP conjecture. Indeed, while it was recently shown by Vojta & Hoyos that strong temporal disorder gives rise to a novel universality class other than DP [46], evidence suggests that if the temporal disorder is comparatively weak, a system can still present critical exponents consistent with DP universality, as in the case of the present model on a neutral fitness landscape but with the time-varying death probability *δ*^τ^ [36]. These exponents quantify the scaling behaviour of the system in the neighbourhood of the phase transition, and values obtained by Scott *et al.* are consistent with the theoretically predicted values for DP [36]. Based on these considerations, we can conclude that the phase transition as a function of *δ*, shown in figure 5, is also consistent with the DP universality class.

In the case of random mating, the dynamic rules of dispersion are no longer short-ranged and thus the third requirement of the DP conjecture is broken; no DP phase transition is observed in this case. This result is also consistent with the body of literature above showing that, in order for emergent clustering to appear, the dynamics must be governed by a local dispersion process and global death [9,12,24–30].

Clusters of the (2 + 1) dimensional DP universality class can be represented as time-dependent bonded genealogical clusters spanning the space-time axes from the first generation to the last generation of the simulation. However, there are also interactions, such as mate choice, between organisms within each generation that go beyond the simple RD process of birth and death. These within-generation interactions (as discussed above) create bonded-clusters of mates on the two-dimensional phenotype space, resulting in another type of phase transition. This transition occurs in the two-dimensional phenotype space, and is unrelated to the spread of the genealogical cluster though time. This phase transition relates to ordinary two-dimensional percolation, in which, once a critical percolation threshold is reached, a cluster of mates can span the space from end to end. Below this threshold, clusters are aggregated and unlikely to span the landscape. This is visually confirmed by population snapshots in figure 4, where the five largest clusters are highlighted. For the values of *µ* and *δ* just entering into the survival regime, the highlighted clusters do not span the morphospace; for values of *µ* and *δ* well into the survival regime, the largest cluster spans the entire morphospace. This implies the existence of a critical value of *δ* for which it becomes possible for a cluster of mates to span the space. The two-dimensional bond percolation phase transition shown here is a continuous, equilibrium phase transition, and thus fundamentally different from the non-equilibrium transition from survival to extinction.

We have also shown that a second type of continuous, spatial percolation phase transition, can occur only after the fitness is increased to *f* = 3 (figure 8). This is an example of a *site percolation* transition. For *f* = 2, no continuous, space-filling, cluster of discs can span the space, even though bonded percolation clusters of mates do span the phenotype space. To be clear in distinguishing the two types of two-dimensional spatial percolation problems at issue, it is important to emphasize that the filling factor is a space-filling measure of continuum percolation, where organisms, defined as discs, can never be closer than *κ* = 0.25 units of each other. Figure 3 illustrates how a connected cluster of mates can span the morphospace at *f* = 2, while the clusters of discs do not. Increasing *f* to 3 and beyond changes the number of diffusing organisms from A → (m + 1)A, where m ≥ 2, thus changing the RD process which drives the system. Changing the RD process from *f* = 2 to *f* = 3 may not remove the system from the DP universality class (T. Vojta 2015, personal communication), yet does change the structure of the DP clusters, because organisms can only sparsely fill the morphospace at *f* = 2, while organisms can pack more closely together for *f* > 2.

The Clark and Evans index *R* characterizes the dispersal patterns of populations based on a nearest neighbour measure [42]. When *R* < 1, the populations are distributed in an aggregated manner; when *R* = 1, they are distributed as if the points were placed randomly on the space; when *R* > 1, the organisms are said to be uniformly distributed within the space. In this sense, the measure is strictly used to identify the patterns of the already dispersed organisms, and does not specifically characterize dispersion owing to the underlying RD process. In other words, it describes the average structure of the population at a moment in time, rather than the DP process of the population across multiple generations. Furthermore, *R* does not distinguish whether a clumped, aggregated population distribution is composed of many small reproductively isolated clusters or one giant cluster. For this reason, it is not a good measure of population disparity. Although the emergent properties of clustering are determined by the type of reproduction the system undergoes, the population distribution as measured by *R* yields a transition from *R* < 1 to *R* > 1 regardless of reproduction scheme. However, it should be noted there are some significant differences in the behaviour of *R* between reproduction schemes (figure 6). The *R* transition occurs in the active phase of the continuous phase transition (figures 6*a*,*b* and 7), but also only for a select range of *μ* values in the random mating case (figure 6*c*).

By quantifying how populations fill the morphospace, the *R* measure directly shows the process by which a species evolves cannot be determined by community pattern alone, as hypothesized by Chave [7]. In other words, the clumping or grouping of morphological characters by nearest-neighbour distance provides little, if any, information about *how* a population evolved. This is highlighted by the fact that the assortative mating and asexual reproduction schemes exhibited many clusters, while the random mating scheme only had one giant cluster on order of the population size, yet all reproduction schemes exhibited an *R* transition, at least for some parameter values (figure 6). Further, the spatially asymmetric birth and death placements, generated via the RD process, allowed for emergent clustering, but do not completely determine organismal dispersal patterns on the landscape. Fig. 3.9 from Henkel *et al.* illustrates how the appearance of DP clusters changes along the phase transition line of the (1 + 1) dimensional Domany-Kinzel model depending on the interplay of the parameters involved [38]. Similarly, the visual appearance of the DP clusters in our model is also likely to change as a function of *δ* as population transition from an aggregated, to a random, to a uniform distribution within the phase transition survival regime. The interesting thing to note here is that, as the changes in distribution are shown to occur in the active phase (figure 7), the different kinds of DP percolation clusters are not restricted to the phase boundary.

The interpretation of clusters, which exhibit reproductive isolation within a given generation, can be roughly analogous to species. However, there are important limitations to this analogy. In a subsequent generation, the descendants of clusters can merge once again, if their offspring are sufficiently close to one another. From this perspective, clusters are more similar to incipient species, where sub-populations might break up the sexual continuum in particular ways or fill niche gaps, but still be able to interbreed [47].

It has been shown by King [40] that independent lineage structures do evolve in this model and that these lineages structure depend heavily on where they are located in parameter space. Near the critical point, lineages exhibited punctuated bursts of evolutionary characters, and macroscopic analysis of branching behaviour (distributions of times to most recent common ancestor) exhibited power-law scaling. This punctuated behaviour was observed for both individual and cluster lineages, indicating that the two levels of organization scale similarly in time.

The model studied here involves evolution on a morphospace only. The addition of a genetic component could be used to impose a more definitive speciation criterion, rendering separate populations as unable to interbreed when they became too genetically dissimilar, like the models developed by de Aguiar [12,48]. It has been shown in such models that the minimal genome size needed for such a speciation event is significantly decreased when spatial restrictions were also imposed on the organisms [48]. By contrast, without a spatial constraint, an infinite genome size is needed [49].

The properties of the DP phase transitions, the ordinary bond percolation properties of clusters of mating groups and the corresponding continuum percolation problem, as well as the *R* transition in the overall dispersion of the organisms regardless of mating scheme all demonstrate the inability of the organisms to completely saturate the morphospace. As noted above, for DP RD dynamics in this model, dispersion was restricted to *f* = 2, and *f* needs to be greater than 2 for a space-filling continuum disc cluster (site percolation) to occur. However, it is possible for a cluster of bonded mates to span the space. This is further consistent with the observation that the maximum *R* value obtained in the simulations for *f* = 2 was 1.42, while a value of *R* = 2.1491 is needed for a maximally filled space [42]. A space-spanning cluster of bonded mates could contain many disc clusters, as shown in figure 3. In other words, the phenotypic characters within a reproductively isolated cluster could cover a widely distributed, and even discontinuous, range.

The sparse filling of phenotype space is consistent with the potential pitfalls (morphological ‘outliers’ occurring within known lineages) discussed by Raup & Gould [33] and Pie & Weitz [9] for classifying lineages based on morphological character alone. Further, as evidenced from soft-bodied creatures of the Burgess Shale, it is possible to have high disparity and low diversity [21,35], indicating that many body plans may exist with groups. This is a significant problem for palaeontologists because much of the taxonomy derived from the fossil record of deep time results from classification via morphological characters. In the context of mass extinction, clades that had high disparity early in their evolution (bottom-heavy clades) were three times more likely to survive a mass extinction, whereas the top-heavy clades were more likely to succumb to one of the ‘big five’ mass extinction events [50]. Yet the potential of populations to recover from mass extinction may depend at least as much on lineage structure as on morphology, and the understanding of the relation between lineage and morphology in this age of the ‘sixth extinction’ [17].

Universality class identification of the non-equilibrium DP transition in this model provides important information about the system dynamics as it moves from survival to extinction. There are non-equilibrium transitions other than DP [38], which exhibit different dynamical signatures (scaling exponents). The particular dynamics of a survival-to-extinction transition may provide tools to investigate biological systems that are nearing collapse, or to predict the conditions under which recovery from mass extinction can occur. Critical dynamics has been used to identify early warning signs of population collapse in various systems [51,52].

DP is a canonical, non-equilibrium transition that can occur in a wide range of systems; systems in other universality classes will have a distinctly different behaviour. In addition to the DP transition, the model investigated here exhibits spatial clustering and an equilibrium bond percolation transition. It is possible that the local clustering dynamics might change significantly if the non-equilibrium transition did not follow the dynamical rules of DP, as is the case when the dynamical rules of offspring dispersal are no longer short ranged (random mating). However, phase transition behaviour in the statistical physics sense has yet to be studied in clustering models like that of Young *et al.* [26]. It is known that short-range interactions are necessary for clustering [9,12,24–30] and also for a DP transition [38]. Beyond this, however, the relationship between specific characteristics of clustering behaviour and the non-equilibrium dynamics of particular phase transitions remains to be explored.

The potential of the present model lies in its ability to track lineages as well as morphological patterns, and to quantitatively characterize the relationship between the two. In the future, continuum percolation may be used to develop a within-cluster diversity measure that could be used to address the relationship between lineage and morphological diversity (e.g. a diversity versus disparity measure), as well as to computationally address the limiting factors in morphospace saturation that contribute to high disparity in early evolution [53] or ontogeny [54]. A number of steps must be taken in order for models of this type to become practically useful to the palaeontological community. These steps will include development of analytical techniques to investigate existing branching patterns, comparison of null models with ones that incorporate selection, simulations of adaptive radiation of both lineages and morphologies during recovery from mass extinction, and the identification of hierarchical relations above the cluster level.

## Data accessibility

All code used in simulation and analysis can be found online at a github repository: https://github.com/DawnMKing.
